# Education of children with disabilities in New Delhi: When does exclusion occur?

**DOI:** 10.1371/journal.pone.0183885

**Published:** 2017-09-06

**Authors:** Parul Bakhshi, Ganesh M. Babulal, Jean-Francois Trani

**Affiliations:** 1 Program in Occupational Therapy, Washington University in St. Louis, St. Louis, United States of America; 2 Department of Neurology, Washington University in St. Louis, St. Louis, United States of America; 3 George Warren Brown School of Social Work, Washington University in St. Louis, St. Louis, United States of America; Universita degli Studi di Perugia, ITALY

## Abstract

**Background:**

In the new Sustainable Development Goal 4, quality of education defined as equity and inclusion alongside traditional learning outcomes, has replaced the narrow goal of access to primary education stipulated in the Millennium Development Goal 2. Since 2000, considerable progress has been made towards improving access to school for children in India, yet questions remain regarding not just children with disabilities’ access and acquisition of basic learning skills, but also completion of learning cycles.

**Methods and findings:**

Between November, 2, 2011 and June 20th 2012, we interviewed 1294 households about activity limitations and functioning difficulties associated with a health problem among all family members using a validated screening instruments, as well as questions about access, retention and barriers to education. We found that vulnerable children, particularly children with disabilities are less likely to start school and more likely to drop out of school earlier and before completing their high school education than non-disabled children, showing that the learning process is not inclusive in practice. The gap is wider for girls, economically deprived children, or children from households where the head is uneducated.

**Conclusions:**

Firstly, in order to fill the existing knowledge gap on education of children with disabilities in line with SDG4, not only is there a necessity for relevant data with regards to learning outcomes, but also an urgent requirement for more innovative information pertaining to relational aspects of learning that reflect inclusion. Secondly, a stronger understanding of the implications of early assessment would further promote equity in education. Finally, research should tackle learning as a complex and dynamic phenomenon. Education needs to fulfil its instrumental value, but must also re-claim its intrinsic value that often gets watered down in the journey from policies to implementation.

## Introduction: The challenge of education in India

As the world’s largest democracy, India has a myriad challenges in order to ensure access to education for all for over 200 million children aged 6 to 13 [1]. It is undisputable that extraordinary progress has been made to improve access of all children to learning systems: in 2002–03 the number of out of school students stood at 32 million, in 2010–11 it was estimated at 2.7 million [1]. A 2014 report from the Annual Status of Education Report (ASER) states that 96.7% of children between ages 6 and 14 living in rural settings are enrolled in schools [2]. In terms of making learning a reality for the most vulnerable sections of society such as girls or scheduled casts and scheduled tribes -scheduled tribes and scheduled casts are official designation used in the Constitution of India for various disadvantaged indigenous people; in the 2011 census both groups represent respectively 16.6% and 8.6% of the population, the progress has been less evident. Experts and commentators agree today that one of the paramount hurdles to cross is the quality of education that children are receiving in schools. In rural schools in 2014, half the children in the 5^th^ standard could not read at the 2^nd^ standard level [1]. According to the 2011 National Census, 1.05% of school going children have a disability (2.13 million); of these 28% (588,000) are not accessing school. More specifically, 44% of children with disabilities not accessing school have multiple and complex forms of activity limitations and functioning difficulties. The census directly asked about disability status (yes/no). Studies show that direct question about a person’s disability status systematically underestimate prevalence of disability [3, 4]. In Delhi more specifically, 32.2% of all disabled children and youth age 5–19 years old– 32.6% of boys and 31.6% of girls- never attended any educational institution. These rates are lower for visual impairment (respectively 16.7% for all, 15.0% for boys and 19.1% for girls), hearing impairment (17.0% for all, 17.3% for boys and 16.6% for girls), mobility limitations (25.4% for all, 25.1% for boys, 25.8% for girls), similar for speech impairment (30.5% for all, 30.9% for boys and 29.9% for girls) and higher for intellectual impairment (51.2% for all, 50.2% for boys and 52.7% for girls) and mental illness (56.2% for all, 56.1% for boys and 56.3% for girls). In this paper, we investigate if the progress made in the last decades in policy of inclusive education through the implementation of Education for All (Sarva Siksha Abhyan- SSA) has succeeded in including vulnerable children for a full primary and secondary cycle of education.

The second section briefly describes the international and national frameworks that shape the political and legal scaffolding of inclusive education in India and discusses the understanding and implementation of inclusive education within the country in the light of a social exclusion lens to help better decipher the complexity of disability. In section three, we present the methods of this study and section four presents the findings about access to education and retention for children with disabilities from a case-control survey carried out in Delhi in 2011. Finally, section five concludes on implications for research and policy.

## Background

### International frameworks and conventions on education

At the international level, education has been stated as a human right. Evidence suggests that it is a powerful means of reducing inequalities, fighting discrimination, promoting social justice and breaking the poverty cycle [5, 6]. The 2010 Global Monitoring Report [7] focused on marginalization and ‘educational poverty’ and its links to well-being and human development [8].

Within the MDGs that ended in 2015, education has been defined narrowly in Goal 2 as access to Universal Primary Education (UPE)[9]. This approach entailed inclusion of all children but failed to emphasize important values for education such as freedom, equality, tolerance and solidarity among others [10]. This approach also ignored ideas of ownership and empowerment as the MDGs in general, and MDG2 more specifically, were determined in the absence of wide and popular debate [11].

The new Sustainable Development Goal 4 (SDG 4) aims to “Ensure inclusive and equitable quality education and promote lifelong learning”, thus recognizing that there is a need to focus on the learning process beyond access [12]. It is more in synergy with the ‘Education For All’ (EFA) movement spearheaded by UNESCO that addresses questions of equity, quality and life-long learning [13, 14].

Inclusive education has diverse definitions. The concept itself is historically associated with disability. The 1994 UNESCO Salamanca Statement tied inclusion understood as respect for diversity to an adaptation of all components of an education system [15]. Recently, the concept of inclusive education has expanded beyond the realms of disability to comprehend issues around gender inequality, ethnic or indigenous minorities, conflict, internally displaced populations, migrants, people living with HIV/AIDS, etc. [16, 17]. Within education, inclusive programmes refer mainly to access. However, inclusion encompasses diversity and equality, and has to be recognised as “*an opportunity for society to examine critically its social institutions and structures (…) and (offer) an opportunity for EFA to begin to make distinctions between ‘moral’ and ‘technical’ reforms*” that need to occur simultaneously [18]. The United Nations Convention for Persons with Disabilities (UNCRPD) constitutes a strong tool to advocate for inclusion in education and calls to State parties to provide resources and reasonable accommodations (article 24) [19]. The new SDG 4 emphasizes this paradigm shift from access towards quality and equity to determine successful educational achievement.

### National educational framework in India

Education in India is managed by the Ministry of Human Resources and Development and does not have a separate political entity. In 1986, the National Policy on Education established compulsory education for all children between the ages of 6 and 14 [20]. The Education For All movement *Sarva Shiksha Abhyan* (SSA) grew to be a national programme following the EFA conference in Dakar in 2000. Although evidence shows that progress was made towards access for vulnerable groups (scheduled casts, scheduled tribes, girls, children with disabilities, etc.) [21], the achievements in terms of quality of learning have been more difficult to grasp, even for the very basic skills of literacy and numeracy [22]. The 2005 Action Plan for Inclusive Education of Children and Youth with Disabilities brought into focus the specific actions required for making education a successful learning experience for this group. In 2009, the Right of Children to free and compulsory education Act (RTE) was passed to ensure compulsory education between the ages of 6 and 14. India signed and ratified the UNCRPD in 2006 [23].

Debates around “inclusion” in general and “inclusive education” in particular have been on-going and unable to grasp and address the complexity of needs of vulnerable groups [24]. Some commentators argue it is a “Western” context concept that still needs to be translated into the various Indian contexts [18, 23]. It is still closely related to disability and several factors make it challenging to identify what inclusive education should entail in the Indian context.

The first roadblock is the lack of reliable, comprehensive and large-scale data with regards to children with disabilities. Until 2011, the national census did not include information pertaining to children with disabilities. The 2011 census comprised of a sole question to screen for disability within households, thus largely underestimating the prevalence of disabilities (2.7%), especially the most stigmatized forms of disabilities. A 2014 report carried out by the Social and Rural Research Institute provides more detailed data: 1.05% of children have a disability (2.1 million) and children with mental or multiple disabilities are disproportionately out of schools: 35.9% and 44.1% respectively [1]. The report does not address factors that maintain children out of learning systems.

There has been wide agreement that teachers’ attitudes, ability and training are crucial to inclusive and equitable education [25, 26]. Research shows that despite positive attitudes towards inclusion, teachers lack knowledge and skills about inclusionary practices and pedagogical approaches. On the other hand, it has been shown that the deep-rooted cultural beliefs with regards to Dalit children lead to discrimination and exclusion of children within the classroom in rural settings [27].Teacher training modules present tools and skills for screening of disability and advocate for early screening and detection essential for successful intervention, especially for multiple and mental forms of disabilities. However, compulsory education set at the age of 6 is a missed opportunity to tackle early childhood education that is crucial for the most vulnerable children with disabilities. Furthermore, training on inclusion pedagogies is not systematic in the teachers’ curriculum.

Finally, policy documents that frame education in India present disability under various labels (autism, hearing, visual, loco-motor, etc.), but ignore compounding factors, such as gender, cast, religion or living in a rural setting, which all require a set of specific actions to ensure meaningful inclusion.

We argue in the present paper that genuine inclusion in education is defined as the opportunities provided to all children, including vulnerable children, to access and remain in the education system to learn knowledge useful to make choices that will lead them to live rewarding, meaningful and productive lives, and trigger social change [28–30]. Such a vision is aligned with the capability approach that distinguishes between instrumental and intrinsic values of education [8, 31]. The SDGs have set the foundation to return the focus to the intrinsic value of education as a basic human right. Impediment to effective inclusion in India translates as an increasing elimination from the education system of vulnerable children, particularly children with disabilities, from primary to high school.

## Method

The study was approved by University College London Research Ethics Committee, UK and Dr Ram Manohar Lohia Hospital Institutional Ethics Committee, India. Written consent was obtained from adult participants and from caregivers/guardians for minor participants.

### Setting and participants

Between November, 2, 2011 and June 20th 2012, we collected information from 1294 households about the disability status of all the family members. We asked about activity limitations and functioning difficulties associated with a health problem among a total of 6779 family members using a validated screening instrument [32], family structure, as well as questions about access and barriers to education. These interviews were part of a large-scale case control study in New Delhi looking at poverty and stigma of persons with mental illness [33]. For the present study we analysed access to education for a sub sample of 2599 individuals between the age of six and 25.

### Survey procedure

Respondents were asked about conditions of access, healthcare, employment, income, livelihood conditions, and social participation of each member or of the household as a whole. Instruments were translated in Hindi with iterative back-translation methods and tested with a pilot survey carried out in October 2011. Investigators trained two experienced supervisors as well as 10 master level students during two weeks. Interviewers were trained on survey concepts and goals (one day), mental illness issues and awareness (one day), interview techniques (eight days including item by item explanation of instruments) followed by review, test and debriefing. Role-play and field practice interviews were organised.

### Statistical analysis

Demographic characteristics for participants were provided using descriptive statistics. Logistics regression was used to determine which demographic predictors influenced access at different levels of education. Scatterplots and histograms were visually checked to ensure normal distribution. To identify the best predictors, the default, force entry method was used for logistic regression. Collinearity diagnostics and residual analysis were used to determine the final model did not violate the assumptions of linearity, independence of errors and multicollinearity. There were three, main outcomes for the regression models: access to primary school using sample with ages ranging from 6–20, access to middle school using a sample with ages 12–25 and access to high school using a sample with ages 16–25. Access to primary school was defined by accessing primary school. Access to middle and high school was indicated by report of educational level. Educational levels ranged from primary school to college. Attendance in concurrent schooling was not available. The primary model used the variable set: age as continuous, gender, religion (Hindu, Muslim, Other), disability (not disabled vs. disabled), asset index (quintiles), head of household gender, head of household education (educated or not) and household size (1–4 members; 5–7; ≥8). Three additional models analysing the impact of disability were calculated. The second model analysed cases where individuals with different types of disability (physical and sensory; mental and cognitive; multiple) were compared to individuals without any disability. The third model analysed cases where individuals with severe disability were compared to non disabled individuals and individuals with mild or moderate disability. The final model analysed cases of disability severity (mild and moderate; severe) and compared them to individuals without any disability. Mild was classified as endorsing one question on the DSQ-34 with any frequency (sometimes, often, always). Moderate was classified as endorsing two questions rated as sometimes or one question rated as often. Severe was classified as at least four questions rated as sometimes, or two questions rated as often or one question rated as always [34]. In these models, all other predictors remained the same but disability was recoded to assess its multidimensional impact on access. Due to a lack of variance, physical and sensory were combined into one category while, learning, behavioural, mood and neurological were combined into another category. Each of the four models were repeated for the three main outcomes. All analyses were conducted in SPSS version 23 (Chicago, IL, USA).

## Results

There were 2,599 individuals who were included and had complete data available for analyses (Table 1). The sample constituted of fairly young, educated persons along with an even distribution among the asset index quintiles. There was a greater proportion of Hindus, male head of household, educated head of household and nondisabled persons. When compared to the 2011 National India Census data for Delhi among ages 5–19, our sample was older (16.88 vs. 11.63), had similar gender distribution (females: 46.9% vs. 45.3%), had smaller portion of those literate below primary (17.2% vs. 29.5%) and had similar religious distribution (Hindu: 86.3% vs. 81.6%). The rates of disability were very different; there was overall more total disability (17.6% vs. 1.1%), across each type, including multiple disabilities (22.3% vs. 14.3%) in our sample compared to the census data.

**Table 1:** Demographics (n = 2599).

	N/Mean	%/SD
Age	16.88	5.56
Female	1220	46.90%
Education		
Not literate	68	2.60%
Literate without formal	20	0.80%
Literate, below primary	448	17.20%
Primary	202	7.80%
Middle	1010	38.90%
Secondary	590	22.70%
Diploma	19	0.70%
Graduate	222	8.50%
Post-graduate	20	0.80%
Asset Index		
1^st^ quintile (poorest)	517	19.90%
2^nd^ quintile	520	20.00%
3^rd^ quintile	542	20.90%
4^th^ quintile	516	19.90%
5^th^ quintile (richest)	504	19.40%
Religion		
Hindu	2243	86.30%
Muslim	222	8.50%
Other	134	5.20%
Head of household Gender, Female	401	15.40%
Household size*	6.5	2.83
Head of household Education		
Below Primary	702	27.00%
Primary or higher	1897	73.00%
Disability		
Nondisabled (n = 2142)	2142	82.40%
Physical (n = 54)	54	2.10%
Sensory (n = 37)	37	1.40%
Intellectual/Developmental Delay (n = 113)	113	4.30%
Behavioral (n = 100)	100	3.80%
Mood ((n = 40)	40	1.50%
Neurological (n = 11)	11	0.40%
Multiple (n = 102)	102	3.90%

Note

* Data on district of residence or geographical location for household was unavailable

Table 2 presents the results of regression models predicting access to primary school. Significant predictors (*p* < .05) included, age, religion, asset and head of household education. Every one-year increase in age was associated with an approximate 3.3% reduced risk of having access to school. Compared to Hindu’s, persons identifying with other, non-Muslim religions were 1.9 times less likely to have access to school. Persons in the fourth asset quintile had 47% reduced risk of having access to primary school compared to the fifth asset quintile or the richest. Persons with a head of household, who was uneducated, were 1.8 times less likely to have access to primary school. Disability as a predictor was statistically significant in all models except the third (*p* < .01). Comparison of persons with severe disability to those without severity did affect access to primary school. Additionally, the second model showed that persons with multiple disabilities were 3.7 times less likely to have access compared to persons without a disability.

**Table 2:** Predicting access to primary school (N = 1745; Age 6–20).

	Model 1^a^		Model 2^b^		Model 3^c^		Model 4^d^	
	OR	(95%CI)	OR	(95%CI)	OR	(95%CI)	OR	(95%CI)
		LL—UL		LL—UL		LL—UL		LL—UL
Age (y)	0.97*	0.941–0.994	0.97*	0.938–0.992	0.97*	0.942–0.995	0.97*	0.941–0.994
Gender (Ref = male)	1.19	0.944–1.505	1.19	0.939–1.499	1.19	0.945–1.507	1.2	0.946–1.509
Religion (Ref = Hindu)								
Muslim	0.99	0.666–1.458	0.99	0.67–1.47	0.98	0.66–1.446	0.98	0.659–1.444
Other	1.92**	1.21–3.057	1.93**	1.211–3.072	1.91**	1.202–3.038	1.92**	1.209–3.056
Disability (Ref = Non disabled)	1.42*	1.049–1.922			1.63	0.971–2.731		
Physical and sensory (61)	NA		1.31	0.745–2.309	NA		NA	
Mental and cognitive (217)	NA		1.12	0.752–1.655	NA		NA	
Multiple	NA		3.65***	1.918–6.96	NA		NA	
Mild and moderate	NA		NA		NA		1.31	0.918–1.864
Severe	NA		NA		NA		1.72*	1.038–2.833
Asset Index (Ref = Richest quintile (5^th^))								
1^st^ quintile	0.99	0.661–1.483	0.97	0.648–1.46	0.99	0.663–1.49	0.98	0.653–1.468
2^nd^ quintile	1.25	0.847–1.83	1.23	0.837–1.814	1.25	0.85–1.838	1.24	0.843–1.824
3^rd^ quintile	1.25	0.858–1.824	1.25	0.856–1.824	1.25	0.858–1.824	1.25	0.855–1.817
4^th^ quintile	0.53**	0.338–0.832	0.53**	0.339–0.838	0.53**	0.335–0.825	0.53**	0.337–0.83
Head of household gender (Ref = Male)	0.84	0.593–1.199	0.82	0.574–1.164	0.85	0.602–1.214	0.85	0.595–1.203
Head of household education (Ref = Educated)	1.77***	1.327–2.369	1.83***	1.365–2.444	1.78***	1.334–2.381	1.78***	1.33–2.375
Household size (Ref = 1–4)								
5–7 members	1.23	0.899–1.693	1.24	0.902–1.702	1.24	0.906–1.706	1.24	0.904–1.703
8 or above	1.27	0.868–1.843	1.27	0.868–1.848	1.26	0.864–1.834	1.27	0.869–1.845
Constant	0.26***		0.27***		0.27***		0.26***	

Note: CI, confidence interval; LL, upper limit; UL, upper limit; OR: odds ratio, significance at the ***(p ≤ 0.001), **(p ≤ 0.01), *(p ≤ 0.05).

Base choice for each outcome is no access to school. The reference category for a predictor is in parentheses.

^A^ This model treated the disability predictor as Not disabled (n = 1467) vs. any disability (n = 278)

^B^ This model treated the disability predictor as Not disabled vs. disabled across different types

^C^ This model treated the disability predictor as Not severely disabled (n = 1668) vs. severely disability (n = 77)

^D^ This model treated the disability predictor as Not disabled (n = 1467) vs. mild and moderate (n = 196) or severe (n = 82).

In the second outcome predicting access to middle school (Table 3), age, asset quintile, head of household gender and education, and household size were significant predictors. Every one-year increase in age was associated with an approximate 6.9% reduced risk of having access. Compared to the richest asset quintile, the second poorest quintile was 2.5 times less likely to have access; while the poorest quintile were 5.3 times less likely to have access to middle school. Persons with a head of household who was uneducated (1.3 times) and female (1.5 times) were less likely to have access to middle school. Compared to small household size (1–4), households with 5–7 members were 2 times less likely to have access, while households with ≥ 8 members were 3.8 times less likely to have access to middle school. Disability as a predictor was not statistically significant in the first or second model. In the third and fourth models, persons with more severe disability were 1.9 and 1.76 times less likely to have access to middle school.

**Table 3:** Predicting access to secondary school (N = 2013; Age 12–25).

	Model 1^a^			Model 2^b^			Model 3^c^				Model 4^d^		
	OR	95% CI		OR	95% CI		OR	95% CI			OR	95% CI	
Age (y)	0.94***	0.90	0.97	0.94***	0.91	0.97	0.93***		0.90	0.97	0.93***	0.90	0.97
Gender (Ref = male)	1.14	0.86	1.50	1.14	0.86	1.50	1.16		0.88	1.54	1.17	0.88	1.55
Religion (Ref = Hindu)													
Muslim	1.45	0.95	2.21	1.45	0.95	2.21	1.42		0.93	2.17	1.44	0.94	2.20
Other	0.89	0.41	0.19	0.89	0.41	1.92	0.89		0.41	1.92	0.88	0.40	1.90
Disability (Ref = Not disabled)	0.98	0.69	1.38				1.93**		1.18	3.14			
Physical and sensory (n = 61)	*NA*	*NA*	*NA*	0.89	0.4	1.92	*NA*		*NA*	*NA*	*NA*	*NA*	*NA*
Mental and cognitive (n = 233)	*NA*	*NA*	*NA*	0.97	0.63	1.48	*NA*		*NA*	*NA*	*NA*	*NA*	*NA*
Multiple (n = 94)	*NA*	*NA*	*NA*	1.06	0.55	2.06	*NA*		*NA*	*NA*	*NA*	*NA*	*NA*
Mild and moderate	*NA*	*NA*	*NA*	*NA*	*NA*	*NA*	*NA*		*NA*	*NA*	0.67	0.43	1.05
Severe	*NA*	*NA*	*NA*	*NA*	*NA*	*NA*	*NA*		*NA*	*NA*	1.76*	1.08	2.88
Asset Index (Ref = Richest quintile (5^th^))													
1^st^ quintile	5.53***	3.34	9.15	5.54***	3.35	9.16	5.32***		3.21	8.80	5.43***	3.28	9.00
2^nd^ quintile	2.57***	1.53	4.32	2.57***	1.53	4.32	2.50***		1.49	4.21	2.53***	1.50	4.26
3^rd^ quintile	1.12	0.65	1.95	1.12	0.65	1.95	1.10		0.63	1.92	1.12	0.64	1.94
4^th^ quintile	0.92	0.51	1.65	0.92	0.51	1.65	0.93		0.52	1.66	0.92	0.51	1.64
Head of household gender (Ref = Male)	1.49*	1.02	2.17	1.48*	1.01	2.16	1.48*		1.01	2.17	1.49*	1.01	2.17
Head of household education (Ref = Educated)	1.39*	1.00	1.93	1.39*	0.52	0.99	1.39*		0.99	1.93	1.40*	1.01	1.95
Household size (Ref = 1–4)													
5–7	1.99***	1.31	3.01	2.00***	1.32	3.03	2.04***		1.35	3.10	2.05***	1.35	3.11
8 or above	3.66***	2.30	5.83	3.68***	2.31	5.87	3.84***		2.40	6.14	3.83***	2.39	6.12

Note: CI, confidence interval; LL, upper limit; UL, upper limit; OR: odds ratio, significance at the ***(p ≤ 0.001), **(p ≤ 0.01), *(p ≤ 0.05).

Base choice for each outcome is no access to school. The reference category for a predictor is in parentheses.

^A^ This model treated the disability predictor as Not disabled (n = 1625) vs. any disability (n = 388)

^B^ This model treated the disability predictor as Not disabled vs. disabled across different types

^C^ This model treated the disability predictor as Not severely disabled (n = 1887) vs. severely disability (n = 126)

^D^ This model treated the disability predictor as Not disabled (n = 1625) vs. mild and moderate (n = 260) or severe (n = 128)

The third and final outcome predicting access to high school (Table 4) showed religion, disability, asset quintile, head of household education and household size are significant predictors. Persons identifying as Muslims were 3.4 times less likely to have access compared to Hindus. Compared to the richest asset quintile, the middle quintile were 1.5 times less likely to have access, the second poorest quintile were 3.8 less likely to have access, while the poorest quintile were 5.9 times less likely to have access to high school. An uneducated head of household was associated with a 2.6 times lower likelihood of having access. Households with 8 or more members had 2.5 times less likelihood of having access compared to smaller households with 1–4 members. Finally, disability was statistically significant in all four models. Persons with a disability were less likely to have access to high school compared to the non-disabled. Persons with multiple disabilities were1.82 times less likely to have access while persons with a severe disability were 1.79 times less likely to have access to high school compared to non-disabled persons.

**Table 4:** Predicting access to high school (N = 1563; Age 16–25).

	Model 1^a^			Model 2^b^			Model 3^c^			Model 4^d^		
	OR	95% CI		OR	95% CI		OR	95% CI		OR	95% CI	
Age (y)	0.97	0.93	1.01	0.96	0.92	1.00	0.96	0.92	1.00	0.96	0.92	1.00
Gender (Ref = male)	1.02	0.81	1.28	1.02	0.81	1.28	1.03	0.82	1.29	1.02	0.82	1.29
Religion (Ref = Hindu)												
Muslim	3.42***	2.11	5.53	3.42***	2.11	5.53	3.47***	2.15	5.61	3.44***	2.12	5.56
Other	1.58	0.94	2.63	1.56	0.94	2.61	1.55	0.93	2.60	1.56	0.94	2.61
Disability (Ref = Not disabled)	1.37*	1.03	1.82				1.75*	1.11	2.76			
Physical and sensory (n = 43)	*NA*	*NA*	*NA*	1.00	0.49	2.01	*NA*	*NA*	*NA*	*NA*	*NA*	*NA*
Mental and cognitive (n = 186)	*NA*	*NA*	*NA*	1.29	0.90	1.84	*NA*	*NA*	*NA*	*NA*	*NA*	*NA*
Multiple (n = 87)	*NA*	*NA*	*NA*	1.82*	1.10	3.01	*NA*	*NA*	*NA*	*NA*	*NA*	*NA*
Mild and moderate	*NA*	*NA*	*NA*	*NA*	*NA*	*NA*	*NA*	*NA*	*NA*	1.19	0.84	1.67
Severe	*NA*	*NA*	*NA*	*NA*	*NA*	*NA*	*NA*	*NA*	*NA*	1.79*	1.13	2.83
Asset Index (Ref = Richest quintile (5^th^))												
1^st^ quintile	5.99***	4.06	8.84	6.06***	4.10	8.95	5.89***	3.98	8.70	5.89***	3.98	8.70
2^nd^ quintile	3.88***	2.69	5.59	3.89***	2.70	5.60	3.86***	2.68	5.56	3.85***	2.67	5.54
3^rd^ quintile	1.56*	1.10	2.23	1.56*	1.09	2.22	1.54*	1.08	2.19	1.54*	1.08	2.20
4^th^ quintile	1.00	0.70	1.44	1.01	0.70	1.45	0.99	0.69	1.43	0.99	0.69	1.43
Head of household gender (Ref = Male)	0.93	0.66	1.44	0.92	0.66	1.30	0.94	0.67	1.32	0.93	0.66	1.31
Head of household education (Ref = Educated)	2.64***	1.97	3.54	2.64***	1.96	3.54	2.68***	1.99	3.59	2.66***	1.98	3.57
Household size (Ref = 1–4)												
5–7	1.01	0.77	1.33	1.02	0.77	1.35	1.00	0.76	1.32	1.00	0.76	1.32
8 or above	2.52***	1.78	3.58	2.57***	1.81	3.65	2.50***	1.76	3.55	2.52***	1.77	3.57

Note: CI, confidence interval; LL, upper limit; UL, upper limit; OR: odds ratio, significance at the ***(p ≤ 0.001), **(p ≤ 0.01), *(p ≤ 0.05).

Base choice for each outcome is no access to school. The reference category for a predictor is in parentheses.

^A^ This model treated the disability predictor as Not disabled (n = 1247) vs. any disability (n = 316)

^B^ This model treated the disability predictor as Not disabled vs. disabled across different types

^C^ This model treated the disability predictor as Not severely disabled (n = 1453) vs. severely disability (n = 110)

^D^ This model treated the disability predictor as Not disabled (n = 1247) vs. mild and moderate (n = 206) or severe (n = 110)

We conducted secondary analyses on the fourth model treating disability as three categories to examine the interaction effects of disability and gender, disability and asset quintile and disability and head of household’s education on access to school. Girls with a disability at any level had similar access to primary and secondary school compared to boys (*p* >.05). However, girls with severe disability were less likely (OR: 2.88; CI: 1.04–8.01; *p* = .04)) to have access to high school compared to boys. The interaction between disability and asset was not statistically significant for access to primary school. However, there was an interaction between persons with severe disability and the third asset (middle; OR: 0.13; CI: 0.03–0.59; *p* = .008) the fourth asset (poor; OR: 0.89; CI: 0.02–0.03 *p* = .008) and fifth asset (poorest; OR: 0.05: CI: 0.01–0.2; *p* < .001) to obtain access to middle school. Increased poverty and disability resulting in reduced access to school. Similarly, persons with a severe disability and in the poorest asset quintile were less likely (OR: 0.13: CI: 0.03–0.57; *p* < .007) to have access to high school. Finally, the interaction effects between disability and head of household education was not statistically significant for access to primary or middle school; however, persons with severe disability with an uneducated head of household were less likely (OR: 0.34; CI: 0.13–0.89: *p* < .029) to have access to high school compared to persons with no disability and with an educated head of household. Finally, we calculated and found 38 households with 2 persons with disability and 2 households with 3 persons with disability. Given that we have over 1200 households, it is very unlikely that there was any household effect associated to the number of persons with disability on the results.

## Discussion

This study aimed to measure the association between disability and access to school at various levels of education, from primary to high school levels in New Delhi, India. Promoting inclusive education is instrumental in improving children with disabilities’ participation and their acceptance, as well as future employment opportunities and social engagement [35–38]. Furthermore, school is a protective factor against potential disability associated with child labour and dangerous working conditions [39]. Finally, having all children in school makes it possible to screen for -and address needs associated with- disability, particularly learning or intellectual disability as shown in other contexts [40, 41].

We found that vulnerable children, particularly children with disabilities are less likely to start school and more likely to drop out of school earlier than non-disabled children and particularly before accessing high school education. Such a deficit in school participation and earlier drop out corroborates similar findings found in other low income countries [38]. We also found that children from poorer households, from a minority religion and who had an uneducated head of household had lower access to schools compared to their counterparts. These results on disproportionate access suggest that while disability is a strong predictor in granting access to school, socioeconomic status, parental education, gender and even household size all impact access to school for children in New Delhi. Despite the free public education available to all citizens, access to education is unequal among many children and further compounded by environmental and socioeconomic factors. The causal link between disability and access to school is not a simple linear relationship but multidimensional and includes multiple factors. Several studies have also found that parents income and level of education influence educational attainment of children [42, 43] or even the decision to send children to school. In the case of children with disabilities, household poverty and parents level of education, but also parents’ attitudes (e.g. beliefs about capacity of children to learn), violence, child labour, disability type (e.g. developmental vs. intellectual vs. acquired from harm), discrimination in the school environment (e.g. bullying, inclusive atmosphere), all impact both access and attendance in school [44–46]. Given the extant literature on schooling and national policies that support access to schools, information about the lack of access and attendance of all children, but especially those with disability is required to understand how such processes of exclusion operate.

### Interpretation of findings

Despite the call for equal education for all children in the Millennium Development Goal 2 and tangible policy efforts towards universal primary education in India, our findings indicate persistent barriers to education for children with disabilities who face the highest injustice in terms of access at all school levels due to existing attitudinal, environmental and institutional barriers [13, 46]. Barriers to education are higher for children with a visible disability–either because of its severity or due to compounding of multiple disabilities. Other studies have shown that access is limited to mainstream schools, particularly for children with severe disabilities and that access tends to be even lower at higher levels of education because they drop out earlier [46, 47]

Barriers to education for children with disabilities are of different nature and thus require comprehensive policies. We identify three interconnected impediments to inclusion. First, children with disabilities might face physical barriers to education, linked to accessibility and transportation availability to reach the school [45]. Second, education systems in most low income countries do not adequately prepare teachers to respond to learner diversity because of the high amount of resources needed to provide personalised learning [48]. Due to lack of funds resulting from economic hardships, children’s specific needs are not covered and this has higher negative consequences for the learning process of children with disabilities assumed to need specific provision including adapted teaching methods, particular equipment, materials or learning context [26, 47, 49, 50]. For instance, Ametepee and Anastasiou (2015) have shown that small amounts of government funding going to special and inclusive education result in poor outcomes for children with disabilities in Ghana. Third, the current state of affairs reflects prejudice and negative attitudes towards the capacity of children with disabilities to be educated alongside other children, as shown by a growing body of literature that points to multiple facets of exclusion. In particular, parents of children with disabilities often believe that they cannot learn because of their impairment. In addition, children with disabilities might face the hostility of other children within and outside the classroom. Furthermore, parents of other children often complain that their own children will not be able to learn and the whole class will be slowed down because of inclusion of children with disabilities. Finally, teachers can at best be concerned with teaching children with disabilities or refusing them in their classroom because they also consider that they should attend special schools.

Exclusion from the education system for children with disabilities increases after completion of primary education in New Delhi. Our findings suggest that this is even more the case for girls with disabilities, for disabled children from the lowest socioeconomic gradients and for children living in households where the head himself or herself is not educated [51]. Girls with disabilities are undergoing double prejudice for being girls–with attached traditional gendered representations, particularly marriage and having children–and being disabled [52–54]. These findings are in line with other studies showing that girls with disabilities in Low and Middle Income Countries may face higher level of attitudinal barriers than other children and do not transfer to secondary schools [55]. Disability and poverty–because of the lack of resources available–are interconnected and mutually reinforcing factors that can lead to deprivation of the capability of education, especially if they are also fuelling social barriers [56–58]. The capability approach, by addressing the issue of justice and equality in education, suggests allocating more resources for persons with disabilities or other vulnerable groups to benefit from an adequate educational capability, which needs to be further examined in the specific context of India. We argue, with other authors, that the capability of education “promote(s) values contributing to flourishing and well-being for all” [59]. This requires that children with disabilities do learn effectively in the classroom [60].

### Moving from the rhetoric of inclusion to analyses of social exclusion: Why children with disabilities don’t learn in class?

Our findings show gaps in inclusion of children with disabilities in Delhi suggesting persistence of structural inequalities within schools in particular, and within societies as wider entities. To address this issue, future research should distinguish between 3 levels of social exclusion proposed by Cohen: (a) the individually-based micro level which focuses on the psychological study of prejudice; (b) the socially-based meso level; (c) the structurally-based macro level [61]. The process of exclusion plays out at various levels; educational assessments, by over-focusing on questions of access, have remained limited to the macro level.

### Moving from focussing on only access to measures of the access-quality dyad

Another important issue to consider for effective inclusion is the risk of “*unfavourable inclusion*” (p. 28), which pertains to unequal terms of social participation [62]. Sen suggests that there are situations where people are in fact included but on unfavourable terms [63]. He further states that the nature of the problem of exclusion and unfavourable inclusion are different and should thus be analysed as distinct phenomenon. In the field of education, the consensus has been on getting children into learning systems. However, our findings show that children with disabilities are not learning enough to remain in school. Shifting the lens to analyse learning processes seems long overdue. In particular the “relational feature” remains elusive in inclusive education programmes’ implementation and more specifically in its assessment. Within this perspective, one can argue that inclusion cannot be assessed only by accounts of access/attendance.

### To conclude: Recommendations for research and policy

Our findings shed light on the exclusion process from school persons with disabilities face in the urban setting of New Delhi. More research is needed to identify causal factors explaining why children and youth with disabilities drop out earlier than their non-disabled counterpart. Further knowledge is required in order to design policies based on evidence pertaining to how exclusion operates within the classroom. Information that goes beyond the habitual educational outcomes like literacy and numeracy and focuses on relational features of educational achievement is needed to meet SDG 4 targets and disentangle processes of exclusion. Currently, culturally sensitive assessment tools of exclusion within the learning system are missing in India specifically, but also in other LICs.

Secondly, focusing on early assessment to detect difficulties before age 6 is crucial. However, the current RTE act does not make education compulsory before the age of 6, which constitutes a missed opportunity for children with the most complex and stigmatized forms of disability.

Thirdly, we advocate for adapted teacher training as a cornerstone of any attempt to achieving inclusion within learning systems. However, the knowledge about pedagogical approaches with regards to various types of disability needs to be complemented with sensitization and awareness training with regards to rights and laws. To date, little is done to tackle the attitudes and beliefs that lead to discriminatory practices within classrooms and schools. Curriculums also need to be scrutinised in the same manner.

Finally, in order to truly grasp and address the challenges of ensuring that children with disabilities not just access but learn in school, researchers must investigate the learning process as a complex and dynamic phenomenon that may reflect negatives attitudes that exist within the wider society. Learning must be targeted and viewed as a central educational achievement. Education needs to fulfil its instrumental value, but must also re-claim its intrinsic value that often gets lost in the translation from policies to implementation.
